# Combined use of the Ilizarov method, concentrated bone marrow aspirate (cBMA), and platelet-rich plasma (PRP) to expedite healing of bimalleolar fractures

**DOI:** 10.1007/s11751-015-0239-x

**Published:** 2015-11-24

**Authors:** Edgardo R. Rodriguez-Collazo, Maria L. Urso

**Affiliations:** Department of Surgery, Presence Saint Joseph Hospital, 2900 N Lake Shore Dr., Chicago, IL 60657 USA; Arteriocyte Medical Systems, 45 South St., Suite 3, Hopkinton, MA 01748 USA

**Keywords:** Mesenchymal stem cell, Tibia, Fracture, Fibula, Nonunion, Comorbidities

## Abstract

Distal tibial and fibular fractures, particularly in patients with comorbidities, heal slowly and have a high incidence of postoperative nonunion and infection. Autologous concentrated bone marrow aspirate (cBMA) and platelet-rich plasma (PRP) increase osteogenic potential of demineralized bone matrix (DBM). The purpose of this case series was to evaluate the efficacy of cBMA, PRP, DBM in conjunction with the Ilizarov fixator as compared to DBM and the Ilizarov fixator alone in expediting fracture healing. Ten patients (mean age 52.9 years) were in the cBMA Group, and 10 patients (mean age 54 years) were in the Control Group. Comorbidities included diabetes, obesity, smoking, and renal disease. Radiographs showed a significant difference in the rate of complete healing in the cBMA Group at 16 ± 1.6 weeks post-surgery as compared to 24 ± 1.3 weeks in the Control Group (*P* < 0.001). No differences were observed between groups in infection rate or nonunions. We conclude that the Ilizarov fixator combined with DBM, cBMA, and PRP expedites fracture healing of the distal tibia and fibula in patients with significant comorbidities.

## Introduction

Distal tibial and fibula fractures can be treated successfully with the Ilizarov fixator [1]. However, complications include contractures, pin track infections, loss of range of motion, and articular damage. Patients who are experiencing soft-tissue loss, comorbidities, and chronic infection increase the complexity of these cases. Patients who are smokers and of advanced age with a high rate of systemic illness are the most difficult to treat because these factors negatively impact the rate of healing and incidence of infection [2, 3]. The challenge surgeons face with these patients is to achieve a stable, well-aligned, mobile and pain-free joint while minimizing the risk of infection and post-traumatic osteoarthritis. The classic Ilizarov technique has several advantages when treating patients with various comorbidities [4]. With this technique, the reduction and fixation of the fracture is performed with minimal soft-tissue exposure and blood loss and the fixation stable enough to allow weight-bearing soon after surgery. The external fixator gives the surgeon the opportunity to adjust the alignment and compress or distract during or after surgery. These factors improve the success rate of the Ilizarov method in fracture repair and contribute a positive effect on patient quality of life [5].

In the treatment for complicated fractures, surgeons may use autologous iliac bone graft (ICBG) to augment healing. Unfortunately, in patients with multiple comorbidities, autologous bone grafting may introduce new complications. These include bleeding, infection, and chronic pain at the donor site. There can be limited bone graft material available for treatment [6]. Since the success of a bone graft is determined by the ability of the grafted tissue to recruit progenitor cells to the injured area to form osteoblasts for healing, alternative methods are needed in compromised patients.

Safe and effective alternative graft materials have become popular for use in patients with significant comorbidities. Demineralized bone matrix (DBM) is donor tissue that has been processed to remove inorganic mineral content resulting in an organic collagen matrix [7]. This leaves DBM with minimal osteoinductive properties. Additional material which improves the osteogenic environment can be obtained from bone marrow aspirate which is concentrated for osteoprogenitor cells. The benefit of bone marrow separation is twofold: Red blood cells are removed since they can interfere with bone formation and healing [6], and concentrated osteoprogenitor cells increase the osteogenic stimulus. This use of concentrated bone marrow aspirate (cBMA) combined with an osteoinductive bone graft substitute (DBM) insures that two of the main properties for bone growth and healing, osteogenesis and osteoinduction, are present with minimal risk and morbidity to the patient. Concentrated BMA provides osteogenic mesenchymal stem cells (MSCs) and hematopoietic stem cells (HSCs). Mesenchymal stem cells secrete various bioactive factors, have inherent differentiation potential, and modulate angiogenic and growth factors in the local microenvironment [8–10]. In addition, MSCs exert anti-inflammatory and immunosuppressive signaling which protect against tissue destruction while facilitating regeneration. Pluripotent hematopoietic stem cells (HSCs) derived from autologous bone marrow may also provide pro- and anti-inflammatory signaling while stimulating the production of angiogenic growth factors via paracrine effects in the treated area [11]. Together, cBMA combined with DBM may expedite healing rates.

The use of platelet-rich plasma (PRP) can augment the healing process of soft tissues following surgical interventions. PRP is a biological intervention prepared as a platelet concentrate after centrifugation of the patient’s whole blood. Through centrifugation, the majority of the red blood cell components and the plasma volume are removed resulting in a post-processed concentration of therapeutic factors (i.e., platelets and white blood cells). Production of PRP also concentrates endogenous growth factors in circulating blood. These factors include platelet-derived growth factor (PDGF), vascular endothelial growth factor (VEGF), transforming growth factor-beta (TGF-β1), epidermal-derived growth factor (EGF), and fibroblast growth factor (FGF) [12–15]. Collectively, PRP stimulates hemostasis, reduces infection, and enhances skin regeneration [16].

The aim of this retrospective case series was to compare the outcome of cBMA and PRP in expediting healing of distal tibial and fibula fractures when used with DBM and the Ilizarov fixator as compared to DBM and the Ilizarov fixator alone.

## Patients and methods

### Study population

Twenty patients (*n* = 20) were included in this retrospective study. Ten patients (*n* = 10, mean age 52.9 years) were in the cBMA Group, and 10 patients (*n* = 10, mean age 54 years) were in the Control Group. Comorbidities included diabetes, obesity, smoking, and renal disease. Ten patients in the cBMA Group had abnormal bone metabolism indicated by vitamin D and calcium deficiency as to seven patients in the Control Group. All patients had closed distal tibial and fibula fractures with a poor soft-tissue envelope.

The study protocol, medical record review procedures, and data analysis were approved by the Presence^SM^ Saint Joseph Hospital Institutional Review Board. Patients were treated between January 2010 and December 2012 with a mean follow-up time of 18 months. Patients were identified by a retrospective review of the electronic medical records. The two cohorts (treatment and control) were identified through the case note review. All identifying data (name, DOB (only age included), demographics) were removed from electronic medical records prior to data analysis.

### Intervention

#### Bone marrow aspiration and concentration

**Fig. 1:**
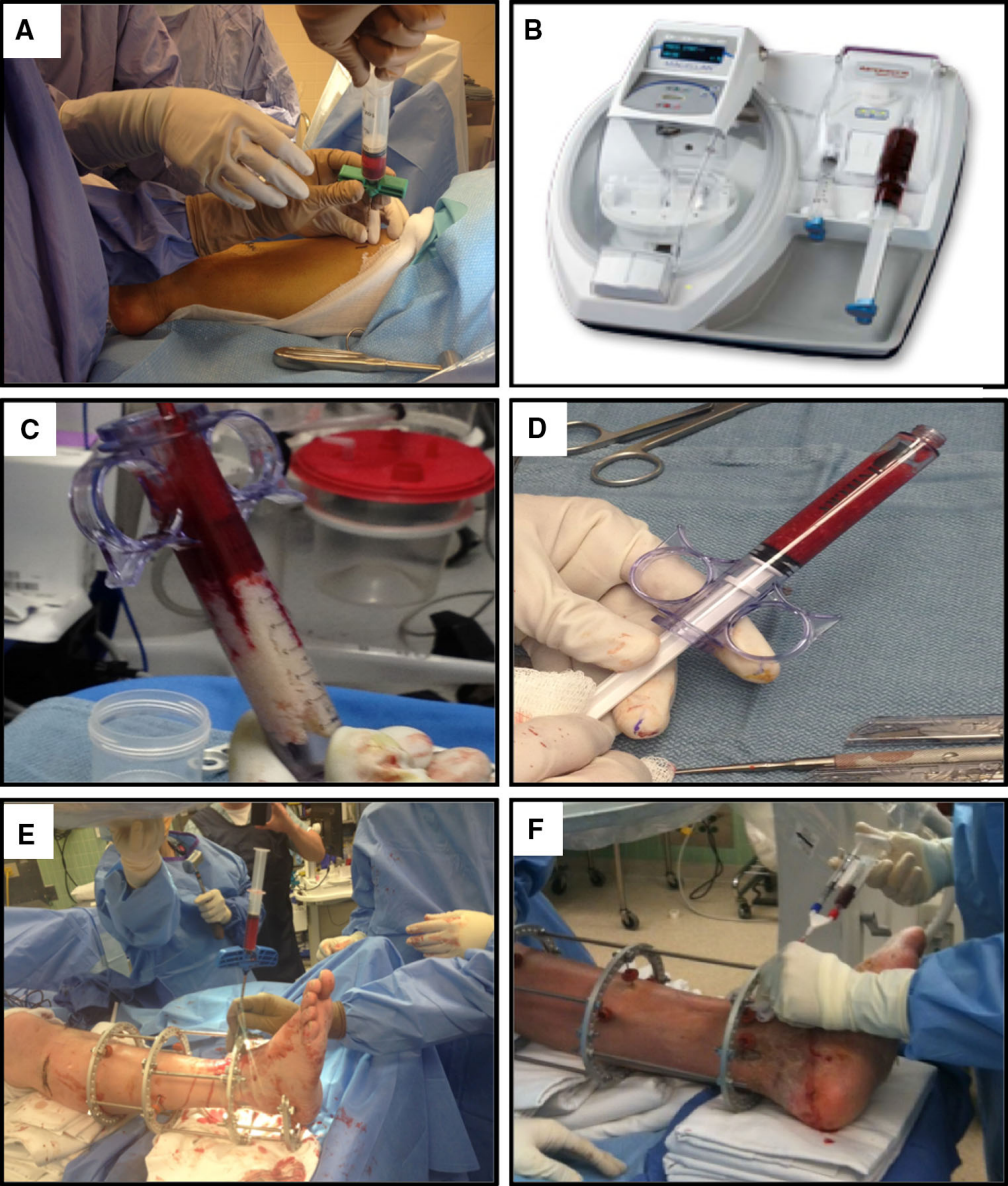
Tissue harvesting and application. **a** Bone marrow aspiration method. **b** Magellan platelet separator. **c** Syringe containing initial mixture of cBMA and DBM. **d** Final implantable mixture of cBMA and DBM. **e** Application of cBMA and graft material to fracture site. **f** Application of PRP using a spray tip cannula

For the 10 patients in the cBMA Group, 26 ml of bone marrow was harvested from the medial aspect of the proximal tibia (Fig. 1a). First, the 30-ml syringe was primed with 4 ml of anticoagulant citrate dextrose solution (ACD-A) (Arteriocyte Medical Systems, Hopkinton, MA). A mallet was then used to advance the 15-gauge Jamshidi needle (Arteriocyte Medical Systems, Hopkinton, MA) until a decrease in resistance was met. The decreased resistance was used to indicate that the needle had entered the marrow cavity. Bone marrow was then drawn back gently (to prevent lysing of the cells) into the 30-ml syringe that was primed with 4 ml of ACD-A. During the marrow draw, if significant resistance was met, the needle was repositioned until the marrow flowed easily. This prevented clotting and unnecessary physical disruption of the aspirate and avoid cell lysis and a reduction in growth factor content of the platelets. The needle was repositioned until a total volume of 30 ml, including the ACD-A, was harvested.

The harvested marrow was then filtered and processed in the Arteriocyte Magellan^®^ system (Arteriocyte Medical Systems, Inc., Hopkinton, MA) (Fig. 1b). The Magellan^®^ is an automatic, dual spin, closed system allowing for safe and rapid bedside concentration of whole blood and bone marrow aspirate. For each case, the Magellan^®^ was customized to obtain a final volume of cBMA of approximately 3 ml from 30 ml of bone marrow aspirate.

#### Phlebotomy and concentration of PRP

For each patient in the cBMA Group, 30 ml of whole blood was drawn from an antecubital vein. The 30-ml syringe was primed with 4 ml of ACD-A before 26 ml of whole blood was obtained from each patient using standard phlebotomy procedures. On reaching the final volume of 30 ml, the syringe was loaded into the Magellan System. The Magellan^®^ was programmed to produce 3 ml of PRP from the 30 ml volume (Fig. 1b).

#### DBM

Integra Accell EVO, which is a DBM and poloxamer Reverse-Phase Medium produced by Integra (Irvine, CA), was used in all patients (cBMA Group and Control Group). This form of DBM provides a high surface area which facilitates binding access for natural bone proteins and a scaffold and signal for new bone formation. The Accell EVO DBM is moldable putty at room temperature which becomes more viscous at body temperature.

Prior to use, the Accell EVO DBM was mixed with the cBMA (cBMA Group only) to achieve desired consistency (Fig. 1c, d). Five cc. of the Accell EVO DBM was injected into fracture sites in both groups (Fig. 1e).

#### Ilizarov technique

**Fig. 2:**
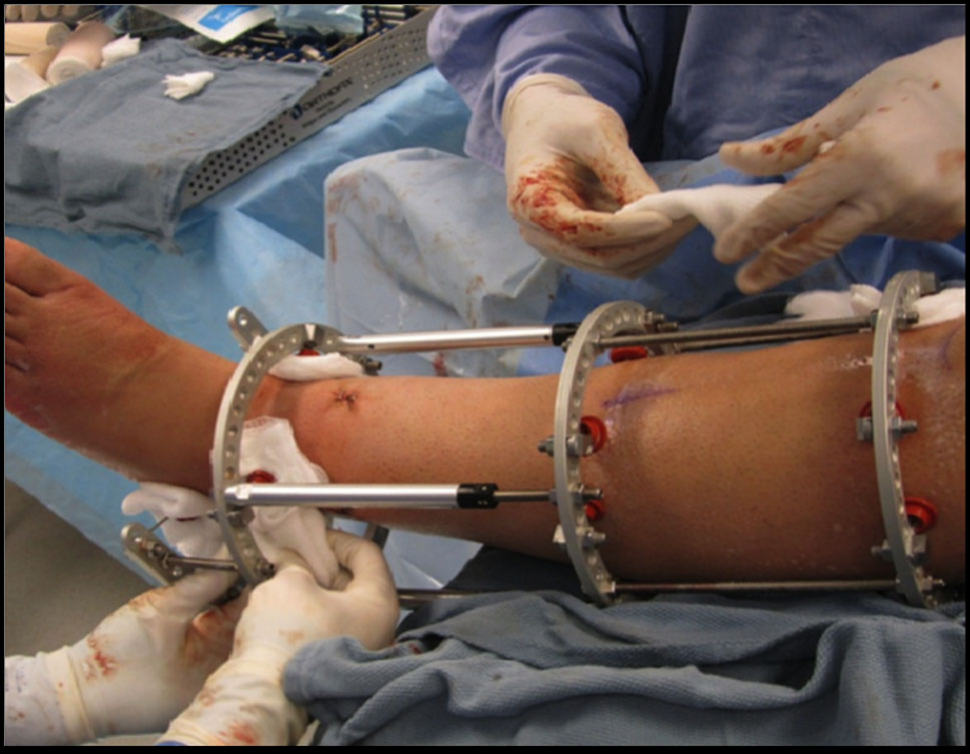
Ilizarov method of fixation. The circular fixator consisted of a pre-constructed frame that consisted of four rings. A proximal reference wire was fixed and tensioned to the most proximal ring. A distal reference wire was fixed directly proximal to the ankle

The circular fixator consisted of a pre-constructed frame that consisted of four rings (Fig. 2). A proximal reference wire was fixed and tensioned to the most proximal ring. A distal reference wire was fixed directly proximal to the ankle. After the bone ends were fixed with opposing olive wires, radiology was used to ascertain alignment of the bone. In cases where the fracture was close to the ankle, the foot was incorporated into the frame to enhance stability and prevent equinus or varus contracture. With the bones were aligned, the cBMA (cBMA Group only) and Accell EVO DBM mixture (cBMA and Control Groups) were injected percutaneously using the 15-gauge bone marrow aspiration needle (Fig. 1e). Viability of the foot was assessed via palpation of the dorsalis pedis and posterior tibial pulses and measurement of oxygen saturation of the hallux. If circulation appeared to be disrupted, the length was adjusted as necessary to return circulation to normal. PRP combined with calcium chloride and thrombin solution was applied using a spray tip cannula at all surgical wound sites in the cBMA Group (Fig. 1f).

Patients were assessed every 2 weeks via X-ray to determine whether the fracture was completely healed and whether frame removal was appropriate. A single observer evaluated the X-rays to determine whether cortices were fully ossified, determined by a sharp outline of the cortical bone. In addition, each patient needed to be able to bear weight on the limb for the frame to be removed. If patients were not healed by week 12, a CT scan was performed. Patients then returned every 2–4 weeks for X-ray or CT scan, respectively, until complete healing was determined. Once the frame was removed, patients were fitted with a patellar tendon brace and instructed to wear the brace for 6 months after frame removal.

### Data collection and statistical analysis

A sample size estimation was performed to identify the number of subjects needed to detect a significant difference in rate of healing between treatment conditions (control vs. cBMA). Sample size estimate ranges were generated using effect sizes and standard deviations at a power of 0.8 and alpha of 0.05 (SigmaPlot v 10.0, Systat Software Inc., Germany). The outcome measures were assessed by physical examination, radiographic examination, and chart review. The mean ± standard deviation was calculated for all measurements reported. To describe the outcomes of the surgical intervention, frequencies and percentages were used for the categorical variables. A one-way analysis of variance (ANOVA) between groups was performed to determine whether there was a significant difference between the rate of healing in the cBMA Group and the Control Group. The critical alpha level was set at 0.05.

## Results

**Fig. 3:**
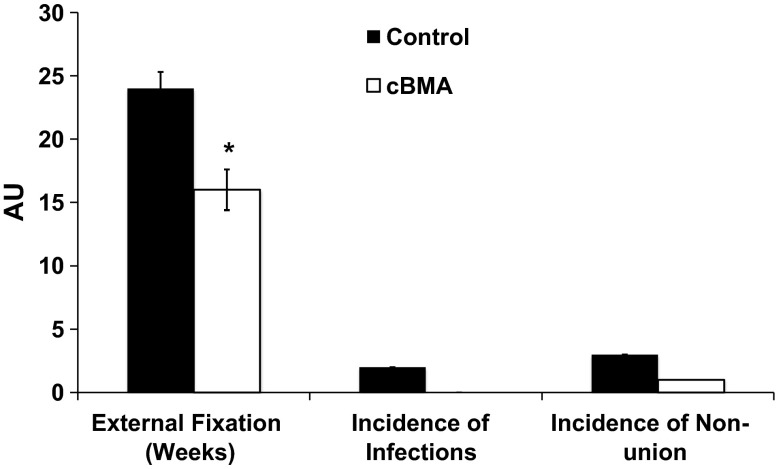
Postoperative outcome measures. There was a significant reduction in the time of external fixation in the cBMA Group. No differences were reported in incidence of infection or nonunion between groups. Data are mean ± SD. *P < 0.001

**Fig. 4:**
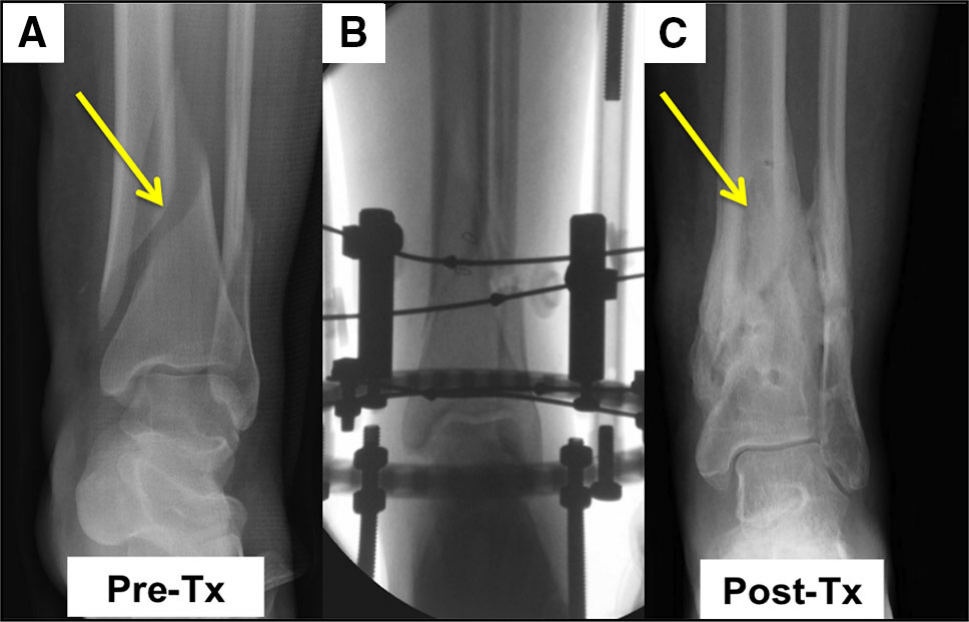
Serial radiographs. **a** Pre-treatment radiograph of a female patient in the cBMA Group with a bimalleolar fracture (*yellow arrow*). **B** Fluoroscopy image of olive wire stabilization of the fracture. **c** Post-treatment radiographs of same female patient at 16 weeks post-procedure. Fracture is healed (*yellow arrow*) (color figure online)

Figure 3 illustrates outcome measures for the cBMA and Control Groups. The mean fixator time was 24 ± 1.3 weeks in the Control Group and 16 ± 1.6 weeks in the cBMA Group (*P* < 0.001). In the Control Group, postoperative radiographs for seven of 10 patients showed complete bone healing at the time of external fixator removal. In the cBMA Group, eight of 10 patients showed complete bone healing when the external fixator was removed at approximately 16 weeks post-surgery (Fig. 4). There were no significant differences between groups in the number of patients with complete bone healing (*P* = 0.6) at the time of fixator removal. Of the two patients in the cBMA Group who experienced delayed union, only one revision was required due to consistent pain. Four weeks post-revision, the patients’ pain had subsided. The second patient did not require a revision, but a percutaneous injection of cBMA was used to augment healing. Both patients healed within approximately 4 months without residual deformity or morbidity. In the Control Group, three patients developed a stiff nonunion without deformity. These patients were braced and were followed for 18 months until complete healing was observed. No surgical revisions were necessary.

Two patients in the cBMA Group experienced superficial irritation as a result of the wires. Antibiotics were not needed in these patients. In the Control Group, two patients developed an infection that required treatment with oral antibiotics. The incidence of infection was not different between groups (*P* = 0.2). No limbs were amputated in this series.

## Discussion

The aim of the author’s strategy in combining cBMA and PRP with the Ilizarov technique and DBM was to decrease the period of external fixation, expedite fracture healing, and diminish the rate of complications, specifically infections. In agreement with our hypothesis, patients treated with cBMA and PRP healed significantly faster than patients in the Control Group. Complications experienced by patients in the cBMA Group were minor, and only one required additional surgery. In the Control Group, three patients experienced nonunion complications, and two experienced skin infections requiring treatment with oral antibiotics. These were not statistically significant, but the trend of fewer skin infections may have arisen from use of PRP at the incision sites in the cBMA Group [17]. Previous reports have evaluated the potent antimicrobial effects of PRP and suggested the combined actions of concentrated levels of leukocytes, platelets, and their derived growth factors have strong bacterial inhibitory effects.

Nonunion is a major challenge to surgeons when treating complex fractures in a patient with multiple comorbidities. Open bone grafting techniques for the treatment for complex fractures have remained unchanged since the early work of Phemister and colleagues [18]. Complications of this technique range from infection, hematoma, and to donor site morbidity. As bone growth is optimal when osteogenic cells are present, techniques that induce osteogenesis while not inducing donor site morbidity in an already-compromised patient are still needed.

This retrospective cohort comparison highlights the benefits of using an autologous source of cells derived from a patient’s bone marrow aspirate to provide an osteogenic milieu. Specifically, mesenchymal cells concentrated from the patient’s bone marrow are multipotent and are able to differentiate into osteoprogenitor cells and osteocytes [19]. Both groups of cells have critical roles in bone remodeling. It is suggested that when osteogenic cells were combined with the inductive properties of the DBM and the stable environment of Ilizarov fixation in this series, healing outcomes were improved and time to union reduced as compared to the Control Group without osteoprogenitor cells. This strategy is of importance in this patient sample as patients here had multiple comorbidities that are associated with poor fracture healing.

There are few reports in the literature evaluating the efficacy of cBMA in conjunction with DBM to promote fracture healing. To our knowledge, this is the first cohort comparison to evaluate the combined use of PRP, cBMA, DBM, and the Ilizarov method in patients with various comorbidities; there was a significant reduction in external fixator time and time to healing. Several reports in the literature suggest the use of PRP on chronic wounds or at postoperative incisions promotes antimicrobial activity, and reduces infection [20]. There was a trend suggested here as no patients in the cBMA Group required oral antibiotics for superficial skin infections.

The limitations of this study include the retrospective nature, the limited number of patients, and the time points at which data were collected. Another factor, currently under investigation in our research group, is the potential variability in the number of progenitor cells (MSCs and HSCs) in a sample of cBMA from each patient. In the population studied, the presence of multiple comorbidities introduces the possibility that patients in the cBMA Group with delayed healing had suboptimal numbers of progenitor cells in their cBMA sample; a larger sample size will be required as well as prior progenitor cell analysis so that patients can be randomized according to progenitor cell number.

In conclusion, the use of DBM saturated with cBMA in combination with the Ilizarov technique results in an 85 % healing rate in approximately 4 months. We conclude this strategy is safe, reliable, and effective with good clinical outcomes for the treatment for complex fractures in patients with significant comorbidities.
